# Supplementary material for: Causal Relationship between Aging and Anorexia Nervosa: A White-Matter-Microstructure-Mediated Mendelian Randomization Analysis
Source: Biomedicines. 2024 Aug 16;12(8):1874. doi: 10.3390/biomedicines12081874 (PMC11351342; doi:10.3390/biomedicines12081874)
Supplement: Supplementary file 1 [file biomedicines-12-01874-s001.zip › Supplementary Figure/Figure S4/Singe SNP plot of MR analysis for TL to ubm-b-1666.pdf]

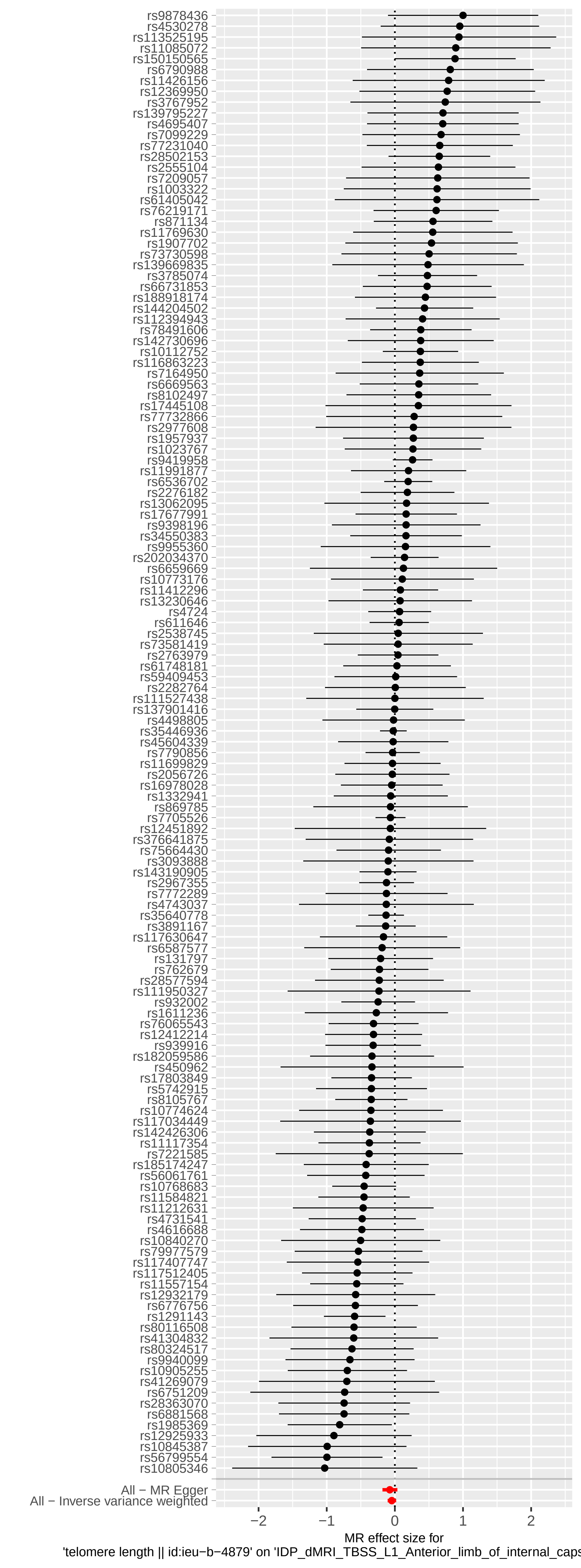
rs9878436
rs4530278
rs113525195
rs11085072
rs150150565
rs6790988
rs11426156
rs12369950
rs3767952
rs139795227
rs4695407
rs7099229
rs77231040
rs28502153
rs2555104
rs7209057
rs1003322
rs61405042
rs76219171
rs871134
rs11769630
rs1907702
rs73730598
rs139669835
rs3785074
rs66731853
rs188918174
rs144204502
rs112394943
rs78491606
rs142730696
rs10112752
rs116863223
rs7164950
rs6669563
rs8102497
rs17445108
rs77732866
rs2977608
rs1957937
rs1023767
rs9419958
rs11991877
rs6536702
rs2276182
rs13062095
rs17677991
rs9398196
rs34550383
rs9955360
rs202034370
rs6659669
rs10773176
rs11412296
rs13230646
rs4724
rs611646
rs2538745
rs73581419
rs2763979
rs61748181
rs59409453
rs2282764
rs111527438
rs137901416
rs4498805
rs35446936
rs45604339
rs7790856
rs11699829
rs2056726
rs16978028
rs1332941
rs869785
rs7705526
rs12451892
rs376641875
rs75664430
rs3093888
rs143190905
rs2967355
rs7772289
rs4743037
rs35640778
rs3891167
rs117630647
rs6587577
rs131797
rs762679
rs28577594
rs111950327
rs932002
rs1611236
rs76065543
rs12412214
rs939916
rs182059586
rs450962
rs17803849
rs5742915
rs8105767
rs10774624
rs117034449
rs142426306
rs11117354
rs7221585
rs185174247
rs56061761
rs10768683
rs11584821
rs11212631
rs4731541
rs4616688
rs10840270
rs79977579
rs117407747
rs117512405
rs11557154
rs12932179
rs6776756
rs1291143
rs80116508
rs41304832
rs80324517
rs9940099
rs10905255
rs41269079
rs6751209
rs28363070
rs6881568
rs1985369
rs12925933
rs10845387
rs56799554
rs10805346
All − MR Egger
All − Inverse variance weighted
−2
−1
0
1
2
MR effect size for
'telomere length || id:ieu−b−4879' on 'IDP_dMRI_TBSS_L1_Anterior_limb_of_internal_caps
